# Infectious complications in children on ventricular assist device: A single-center retrospective cohort study

**DOI:** 10.1371/journal.pone.0335841

**Published:** 2025-11-06

**Authors:** Raphael Seiler, Nehle Speckmann, Camilo Jose Hernandez Toro, Dörte Huscher, Felix Berger, Joachim Photiadis, Mi-Young Cho, Katharina Schmitt, Oliver Miera

**Affiliations:** 1 Deutsches Herzzentrum der Charité, Department of Congenital Heart Disease – Pediatric Cardiology, Augustenburger Platz, Berlin, Germany; 2 Institute of Biometry and Clinical Epidemiology, and Berlin Institute of Health, Charité-Universitätsmedizin Berlin, corporate member of Freie Universität Berlin and Humboldt-Universität zu Berlin, Charitéplatz, Berlin, Germany; 3 Department of Pediatric Respiratory Care, Immunology and Intensive Care Medicine, Charité – Universitätsmedizin Berlin, Berlin, Germany; 4 Clinical Trial Office, Charité – Universitätsmedizin Berlin, Berlin, Germany; 5 Deutsches Herzzentrum der Charité, Department of Congenital and Pediatric Heart Surgery, Augustenburger Platz, Berlin, Germany; Scuola Superiore Sant'Anna, ITALY

## Abstract

**Background:**

Mechanical circulatory support with ventricular assist device (VAD) is a life-saving therapy in children with end-stage heart failure. Infections are a major problem in VAD-therapy and may lead to significant morbidity and mortality. The aim of the study was to evaluate possible risk factors for superficial percutaneous lead/canula infections (SI) and to determine their impact on overall outcome during VAD-therapy.

**Methods:**

Single center, retrospective analysis of infectious complications in 70 consecutive children supported on a VAD (58 Berlin Heart EXCOR Pediatric pulsatile flow pump and 12 Heartware continuous flow pump). Cox proportional hazard models with SI, bloodstream infections, and stroke as outcome, as well as a competing risk model for device weaning, death, and heart transplantation were used to identify risk factors in the study population.

**Results:**

SI were documented in twelve out of 70 children (17%). The event rate for SI was 5.86 per 100 patient months [95%CI 3.03–10.23] with a median time to SI of 109 days [IQR 66–163]. The occurrence of SI was mostly in patients with longer support times while the underlying diagnosis and previous thoracotomies had no impact on the number of SI. Further, children older than three years of age had a higher risk for SI (HR 2.67; 95% CI 1.09–6.57, p = 0.032). SI were not associated with the risk of bloodstream infections (HR 1.27; 95%CI 0.44–3.67, p = 0.656) or death (HR 0.32; 95% CI 0.06–1.80, p = 0.194).

**Conclusions:**

SI occurred frequently during VAD therapy in children, without leading to a higher rate of bloodstream infections or mortality. Further, being above three years of age at VAD implantation was associated with increased risk to develop SI.

## Introduction

Therapy with ventricular assist devices (VAD) has led to a remarkable reduction in mortality amongst children awaiting heart transplantation during the last decades [1,2]. About 30% of all pediatric patients are treated with mechanical circulatory support while awaiting heart transplantation [3]. However, infections remain a major problem during long-term mechanical circulatory support.

In pediatric cardiac centers worldwide, as well as in our study setting, two types of VADs are predominantly used for long-term mechanical support in children: paracorporeal pulsatile devices (Berlin Heart EXCOR) and implantable continuous flow pumps (to date HeartMate 3, in the past HeartWare HVAD). The Berlin Heart EXCOR Pediatric is a paracorporeal, pulsatile flow system (PF) [4], suitable for left ventricular or biventricular mechanical support. Continuous flow (CF) VADs are fully implanted systems, connected with a driveline to the extracorporeal driving unit through the abdominal wall [5].

Despite improved clinical management, complications remain frequent during VAD-therapy [6,7]. In pediatric patients, device malfunction, bleeding, thromboembolic events, and infections are the most prevalent complications [6,8]. Cannulas or drivelines of the devices are passed through the skin, which makes them prone to infections. Goldstein et al. reported that “Percutaneous site and/or pocket infections” are the most frequent adverse event in adults on VAD therapy with adverse effects on survival [9]. Similarly, Auerbach et al. showed that in the PediMACS cohort (Pediatric arm of INTERMACS, the interagency registry of mechanically assisted circulatory support) 20% of pediatric patients had an “External pump component infection” [10]. Life threatening systemic infections, such as sepsis, endocarditis, or mediastinitis may be triggered by local infections [11,12]. Additionally, systemic infections may be associated with thromboembolic events in adults [13], causing subsequent severe neurological damage [14,15]. However, the data on infections is still heterogenous, due to variable definitions and small study cohorts in children. Even less is currently known about local infections of the cannulas and/or driveline, according to the actual definition referred to as superficial percutaneous lead/canula infections (SI) [16]. To date, the incidence of SI, its correlation to the duration of VAD therapy, and its association with possible risk factors such as VAD-type, patient age, underlying diagnosis and presence of previous thoracotomies remains elusive. Moreover, to the best of our knowledge, the impact of SI on bloodstream infections (BSI) and thromboembolic events in children has not been described in the literature yet.

Therefore, we conducted this retrospective single-center study to gain deeper insights into the incidence of SI, associated risk factors, and the impact of SI on BSI, thromboembolic events, and overall clinical outcomes in pediatric patients supported with a VAD.

## Patients and methods

### Study setting and recruitment

**Fig 1:**
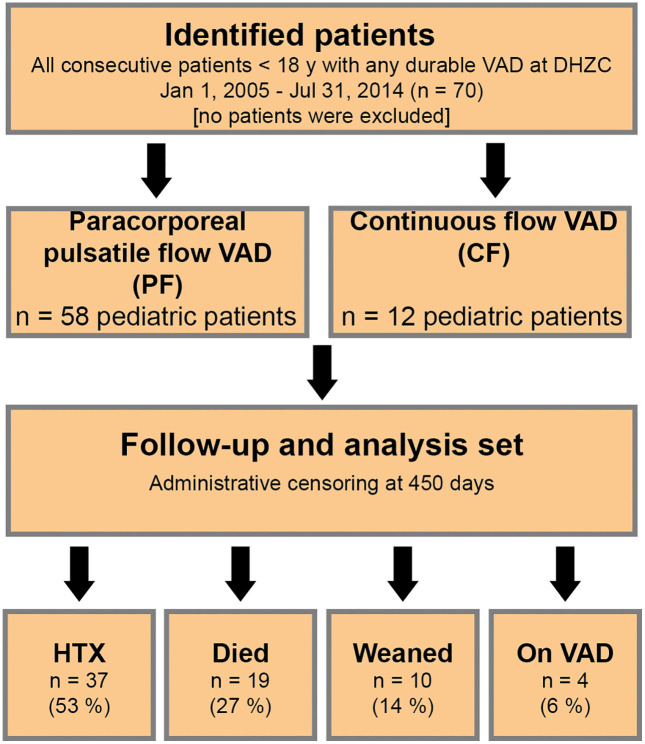
Study flow of participants. VAD = Ventricular assist device; HTX = Heart transplantation, DHZC = Deutsches Herzzentrum der Charité.

This single-center retrospective cohort was conducted at the Deutsches Herzzentrum der Charité (DHZC), a tertiary referral center for pediatric cardiology and congenital heart disease with pediatric mechanical circulatory support and transplant programs. We identified all consecutive patients <18 years of age who received durable VAD support at our institution between January 1, 2005 and July 31, 2014 (Fig 1). To reflect routine care and maximize generalizability, no exclusion criteria were applied.

Data were abstracted from clinical records 05/2015–12/2015 and pseudonymized before analysis; all data were obtained during clinical routine care under ethics approval by the Charité-Universitätsmedizin Berlin (Germany) Ethics Committee (No: **EA 2/050/15)**. According to the approval of the Charité–Universitätsmedizin Berlin Ethics Committee and in compliance with the Declaration of Helsinki, no additional written or oral consent was required. Device types in this cohort included paracorporeal pulsatile-flow systems (Berlin Heart EXCOR®) and implantable continuous-flow pumps (HeartWare®). Follow-up for analyses was administratively censored at **450 days.** The primary outcome of the present study was the development of SI, while secondary outcomes included bloodstream infection (BSI), stroke, heart transplantation, successful weaning from VAD, or death.

**Fig 2:**
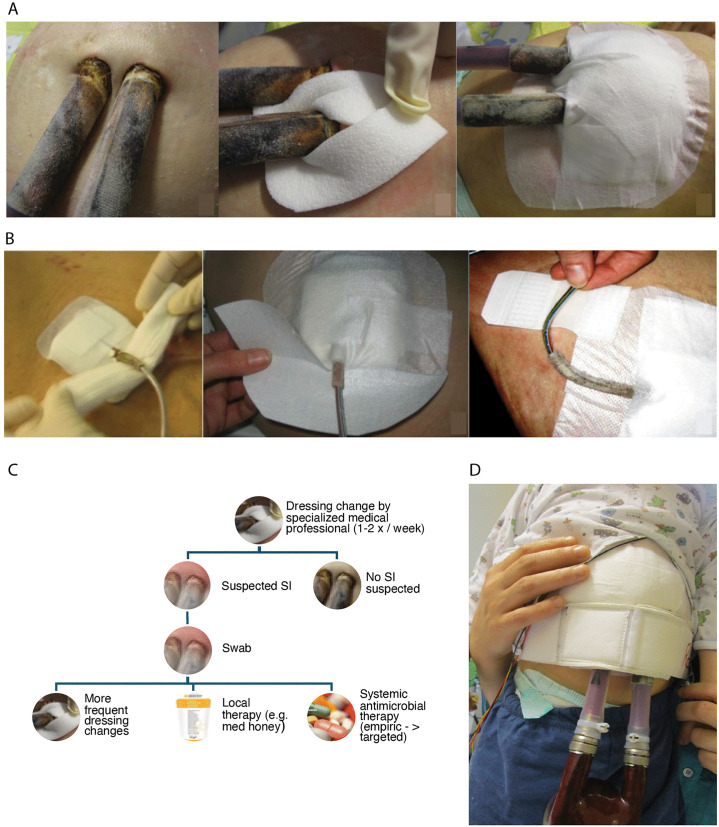
Wound dressing in VAD patients. Representative images for routine wound dressing change of a paracorporeal pulsatile VAD system **(A)** and the driveline of an intracorporal continuous flow VAD system **(B)**. Routine workflow to prevent SI in VAD patients at Deutsches Herzzentrum der Charité **(C)**. Immobilization belt **(D)**. VAD = Ventricular assist device; SI = Superficial percutaneous lead/canula infections.

Data on SI obtained during dressing changes were analyzed. The skin surrounding the percutaneous lead or cannulas was inspected by specialized medical professionals at least once a week and a wound swab was taken for analysis when there was clinical evidence of infection such as drainage, erythema, pain, fever, or leukocytosis (Fig 2). This was performed by educated wound managers with expertise in care of children, small team of two nurses, frequency was higher if wound not found to be completely normal, up to daily changes. Consultation with VAD physician and infections disease specialist if clinical suspicion of infection.

For the diagnosis of superficial or deep percutaneous lead infections, infection of external surfaces, blood-contacting surfaces and non-MCS related infections like mediastinitis or blood stream infection the updated definitions of adverse events according to the consensus statement of the mechanical circulatory support academic research consortium were applied [16]. As there is no adequate definition for superficial canula infections in paracorporeal VADs, we have used the definition for lead infection accordingly.

To compare incidence of SI and evaluate possible risk factors, patients were categorized as follows: age 0–36 months or older than 36 months; diagnosis cardiomyopathy/myocarditis or congenital heart disease; VAD-type Berlin Heart EXCOR^®^ Pediatric (PF LVAD, PF RVAD or PF BVAD) or Heartware System (CF LVAD); secondary clinical outcomes were defined as: BSI, stroke, heart transplantation, death on VAD, weaned from VAD, or still on VAD at the end of data collection.

### Statistical analysis

#### Descriptive statistics.

Continuous variables were described by mean and standard deviation or median and interquartile range (IQR, 1st quartile to 3rd quartile). Categorical variables were described by absolute and relative frequencies. All analyses considered follow-up periods longer than 450 days as censored at 450 days. Kaplan Meier curves for SI with respect to different risk factors were generated. Cox proportional hazards (CPH) models were fitted on the data for the events SI, BSI, and stroke. For all models, initial variable selection was based on medical expectation. The VAD-type, patient sex and age group (Toddlers: 0–36 months; non-toddlers: older than 36 months), underlying diagnosis, and presence of previous thoracotomies at date of device implantation were considered time-invariant covariates. Given that SI never occurred before the first instance of stroke, SI was not included in the CPH model for stroke. In the CPH model for BSI the presence of a SI was considered as time-dependent covariate, and once a patient suffered from SI, the wound infection was considered present until the end of follow-up. To allow the inclusion of recurrent events of a single patient in the analysis, the approach described by Andersen and Gill was used to model the recurrence of BSI and of stroke [17]. This approach assumes that recurrent events on an individual are explained only by the covariates included in the model and it is commonly applied when the interest is in the overall effect of the covariates on the risk of occurrence of a recurrent event and not in the effects on the risk of each individual recurrence.

The different clinical outcomes (weaning from VAD, heart transplantation, and death) were modeled using a competing risks CPH model. VAD-type, patient sex and age group at device implantation, presence of previous thoracotomies at date of device implantation, underlying diagnosis, SI, BSI, and stroke were considered time-invariant covariate [17]. A step wise backwards variable selection procedure was applied [18,19]. The best model was updated in each iteration of the variable selection process only if a difference in Akaike Information Criterion (AIC) [18] of at least 2 units was found for the new selection step [19]. We did not perform any adjustments for multiple comparisons given the exploratory nature of the study. Thus p-values < 0.05 were considered statistically significant. All analyses were performed on complete observations given that there were no missing values present in the data. All statistical analyses were conducted using R (version 4.3.2) (http://www.r-project.org/). Data was prepared using packages from the ‘tidyverse’ (version 2.0.0). The ‘tableone’ package (version 0.13.2) was used to generate summary statistics, ‘epiR’ (version 2.0.76) to calculate incidence rates and their confidence intervals, ‘sjPlot’ (version 2.8.16) to generate regression model summaries, ‘survival’ (version 3.7.0) to fit CPH models, ‘mstate’ (version 0.3.2) to fit competing risk CPH models, ‘foresplot’ (version 3.1.3) to generate forest plots, and ‘survminer’ (version 0.4.9) to generate Kaplan-Meier plots [20–27]. For this article we followed the STROBE reporting guidelines.

## Results

### Patient cohort

In total, 70 pediatric patients (28 female; 40%) treated with VAD were included. Patient characteristics are summarized in **Table 1**. Median age at VAD implantation was 4.5 years [IQR 0–12 years]. The total number of support days was 8735 with a median support time of 56 days [IQR 26–125 days]. A total of 58 patients (83%) were supported with a pulsatile flow PF VAD and 12 patients (17%) with continuous flow CF VAD.

**Table 1:** Demographic data and clinical outcome.

	*All patients (n = 70)*	Superficial percutaneous lead/canula infections (n = 12)	Without Superficial percutaneous lead/canula infections (n = 58)
Sex			
* Female (n)*	28 (40%)	4 (33%)	24 (41%)
* Male (n)*	42 (60%)	8 (67%)	34 (59%)
Weight (kg)	22.7 [21.3]	24.9 [24.09]	22.2 [20.89]
Age (years)	4.5 [0-12]	5.5 [0-9]	3 [0-12]
* 0–36 months (n)*	33 (47%)	4 (33%)	29 (50%)
* > 36 months (n)*	37 (43%)	8 (67%)	29 (50%)
VAD-type			
*Berlin Heart EXCOR*^*®*^ *(PF, n)*			
* LVAD/RVAD (n)*	46 (66%)	7 (58%)	39 (67%)
* BVAD (n)*	12 (17%)	3 (25%)	9 (15%)
* HeartWare*^*®*^ *System (CF, n)*	12 (17%)	2 (17%)	10 (17%)
Time on VAD (days)	56 [26-125]	320 [177-416]	42 [24-77]
Diagnosis			
* Cardiomyopathy or myocarditis*	55 (79%)	11 (92%)	44 (76%)
* CHD*	15 (21%)	1 (8%)	14 (24%)
Blood stream infection (n)	17 (23%)	9 (75%)	8 (14%)
Prior sternotomy (n)	16 (23%)	2 (17%)	14 (24%)
Stroke (n)	16 (23%)	3 (25%)*	13 (22%)
Outcome			
* HTX (n)*	37 (53%)	6 (50%)	31 (53%)
* Died (n)*	19 (27%)	2 (17%)	17 (29%)
* Weaned from VAD (n)*	10 (14%)	1 (8%)	9 (16%)
* Still on VAD (n)*	4 (6%)	3 (25%)	1 (2%)

PF = pulsatile flow; CF = continuous flow; CHD = congenital heart defect; LVAD = left ventricular assist device; RVAD = right ventricular assist device; BVAD = biventricular assist device; HTX = heart transplantation. Continous variables are shown as *mean* [standard deviation] or *median* [Q1, Q3]; categorial variables as *n* (percentage); *Stroke occurred before superficial percutaneous lead/canula infection.

A total of 37 patients were successfully transplanted (52.9%), ten patients were weaned from VAD (14.3%), 19 patients died during VAD therapy (27.1%), and four patients were still on a VAD (5.7%) at the end of the observation.

### Superficial percutaneous lead/canula infections

**Fig 3:**
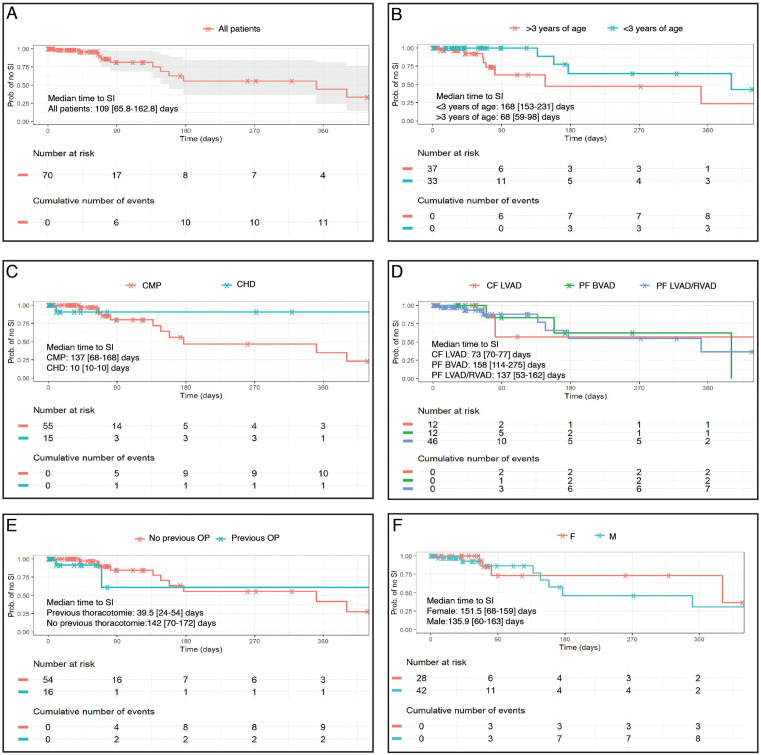
Superficial percutaneous lead/canula infections over time. Time to first superficial percutaneous lead/canula infections are displayed for all patients **(A)**, separated by age group **(B)**, by underlying diagnosis **(C)**, by VAD type **(D)**, by presence of previous operation at VAD implantation **(E)** and by sex **(F)**. SI = superficial percutaneous lead/canula infections; CMP = Cardiomyopathy: CHD = Congenital heart defect; CF LVAD = Continuous flow left ventricular assist device; PF BVAD = Pulsatile flow biventricular assist device; PF LVAD/RVAD = Pulsatile flow left/right ventricular assist device; OP = Operation; F = Female; M = Male.

Twelve out of 70 patients met the criteria for SI (17%; 5.86 infections per 100 patients-months; 95%CI 3.03–10.23) as summarized in **Table 1**. Early infections, defined as infection within the first three months after implantation, and exclusively late infections occurred in six cases each (early: 5.03 infections per 100 patient months; (95%CI 1.85–10.95); late: 3.09 infections per 100 patient months; (95%CI 1.13–6.73)). Median time to infection was 109 days [IQR 66–163] (Fig 3), while patients who did not develop SI either stopped the VAD support much earlier or observation ended due to censoring or death (median time on VAD 42 days; [IQR 24–77]). No patient under one year of age suffered from SI.

In eleven SI cases underlying diagnosis was cardiomyopathy or myocarditis (92%) and in one SI case congenital heart defect (8%). Seven patients who developed SI were supported by PF LVAD or PF RVAD (58%), three patients by PF BVAD (25%) and two patients by CF LVAD (17%). Two of the patients who developed SI had a median sternotomy prior to VAD implantation (17%). One out of 70 patients developed a localized non-MCS device infection at a percutaneous endoscopic gastromy tube leading to SI with the same pathogen and subsequently causing sepsis. One out of 70 patients developed mediastinitis after thoracotomy followed by substernal abscess formation. Details of all twelve patients who had a SI are shown in **Table 2**. In eight patients, one pathogen was detected, while in four patients more than one pathogen was found. The most common pathogen was S*taphylococcus epidermidis*, followed by candida species. We did not detect oxacillin resistant staphylococcus or vancomycin resistant enterococcus.

**Table 2:** Characteristics of the 12 patients with superficial percutaneous lead/canula infections.

*Sex*	Age (y)	Diagnosis	VAD-type	Days on VAD	Days without Infection	Stroke	Microbiology			Lab			Temp	Outcome
							Swab	Blood culture	Drug (i.v.)	WBC/nl	nGr > 10%	CrP (mg/l)		
m	14	CMP/MC	PF	124	41		C. albicans	Staph. aureus	Vancomycin (d 73–84)	1.5	no	10	37.4	Weaned
f	9	CMP/MC	PF (BiVAD)	199	69		Steno. maltophilia	Steno. maltophilia	Ceftazidim + Ampicillin (d 74–100)	10.1	no	0	37.8	HTX
m	7	CMP/MC	PF	432	351		E. faecium, C. albicans/glabrata	Staph. epidermidis	Voriconazol + Cefazolin (d 356–413)	17.5	yes	3.2	38.4	Death
m	4	CHD	PF	26	10		C. albicans	None	Fluconazol (d 4–25)	7.6	no	15.2	38.1	Death
f	9	CMP/MC	PF	128	65		C. albicans Strep. mitis	Serratoa macescens	Tazobactam (d 65–74)	14.0	no	11.2	37.6	HTX
f	6	CMP/MC	CF	351	81		Proteus mirabilis	Staph. aureus	None	9.12	no	1.4	n.a.	HTX
m	5	CMP/MC	PF	193	147		Staph. hominis	Staph. hominis*	None	9.98	no	1.9	38.8	HTX
m	1	CMP/MC	PF (BiVAD)	478	158	X*	Staph. Epidermidis, E.faecalis, Staph. aureus	Staph. Epidermidis, E.faecalis, Staph. aureus	Tazobactam + Linezolid + Meropenem (d 158–175)	17.0	no	9.5	38.2	HTX
m	14	CMP/MC	CF	793	66	X*	Staph. epidermidis, Staph. aureus	Staph. aureus	Tazobactam (d 66–79)	15.5	no	20.4	39.0	Still on VAD
f	1	CMP/MC	PF (BiVAD)	411	391	X*	Staph. epidermidis	None	None	6.7	no	0.16	n.a.	HTX
m	0	CMP/MC	PF	382	137		Staph. epidermidis	Staph. epidermidis	None	5.85	no	n.a.	37.4	Still on VAD
m	0	CMP/MC	PF	389	177		Staph. epidermidis	None	None	12.5	no	0.3	38.0	Still on VAD

m = male; f = female; CMP = cardiomyopathy; MC = myocarditis; CHD = congenital heart disease; PF = pulsatile-flow VAD; CF = continuous-flow VAD; BiVAD = biventricular assist device; C. = candida; Steno = stenotrophomonas; E. = enterococcus; Staph. = staphylococcus; Strep. = streptococcus; d = day; WBC = White blood cell count; nl = nanoliter; nGr = neutrophile granulocyte; CrP = C-reactive protein; Temp = temperature; HTX = heart transplantation; *before occurrence of SI.

### Risk factors for superficial percutaneous lead/canula infections

**Fig 4:**
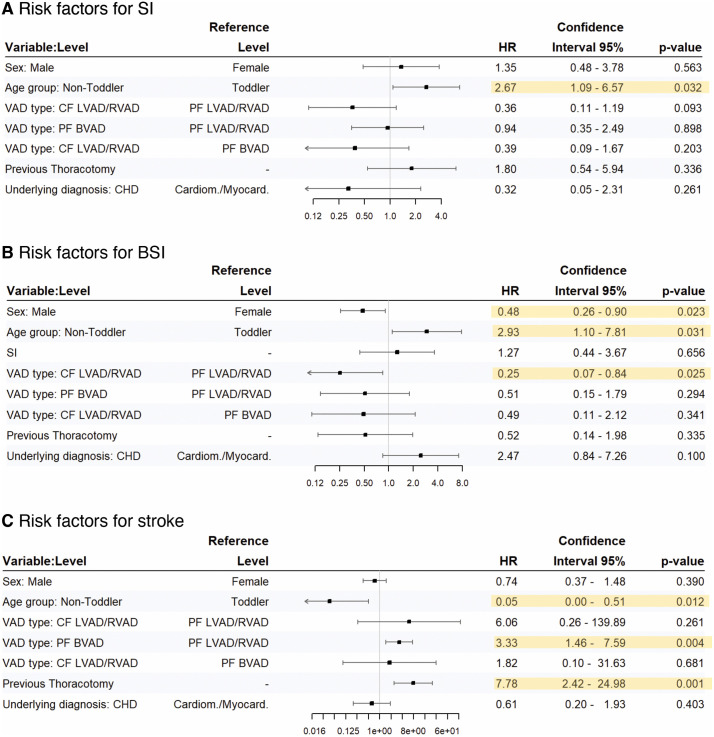
Risk factors (Cox proportional hazard models). Hazard ratios for possible risk factors to develop a superficial percutaneous lead/canula infection are displayed in **(A)**, a BSI in **(B)** and a stroke in **(C)**. SI = superficial percutaneous lead/canula infections; Toddler = < 3 years of age; non-Toddler >3 years of age; Cardiom./Myocard. = Cardiomyopathy/Myocarditis; CHD = Congenital heart defect; CF LVAD = Continuous flow left ventricular assist device; PF BVAD = Pulsatile flow biventricular assist device; PF LVAD/RVAD = Pulsatile flow left/right ventricular assist device. BSI = Bloodstream infection.

An age above 3 years at VAD implantation appears to increase the risk of SI occurrence (HR 2.67 95% CI 1.09–6.57, p = 0.032) while support with a CF LVAD also suggests a protective trend, though this did not reach statistical significance (HR 0.36 95% CI 0.11–1.19, p = 0.093). We could not detect any difference regarding the frequency of SI between PF LVAD/RVAD and PF BVAD devices, underlying diagnosis and the presence of previous thoracotomies (Fig 4).

### Risk factors for bloodstream infections

Seventeen patients (24%) developed a total of 36 BSI (13.73 infections per 100 patient months; 95%CI 9.61–19.00). In seven BSI cases, the same pathogen was identified in a previous SI. Nevertheless, our model did not reveal SI as a relevant risk factor for BSI (HR 1.27; 95% CI 0.44–3.67, p = 0.66) (Fig). Male sex was associated with a lower hazard of BSI (HR 0.48; 95% CI, 0.26–0.90; p = 0.023), whereas age > 3 years was associated with a higher hazard (HR 2.93; 95% CI, 1.10–7.81; p = 0.031).

Patients treated with a CF VAD device were less likely to develop a BSI compared to patients on a PF VAD (HR 0.25; 95% CI 0.07–0.84, p = 0.025). Presence of previous thoracotomies and underlying diagnosis were not linked to the risk of BSI.

### Risk factors for ischemic/ hemorrhagic stroke

During the study period 16 patients (23%) were diagnosed with a total of 18 ischemic or hemorrhagic stroke events (6.86 events per 100 patient months; 95% CI 4.07–10.85). None of them had a previously reported SI, therefore SI and stroke were not correlated in our study cohort.

### Outcome

**Fig 5:**
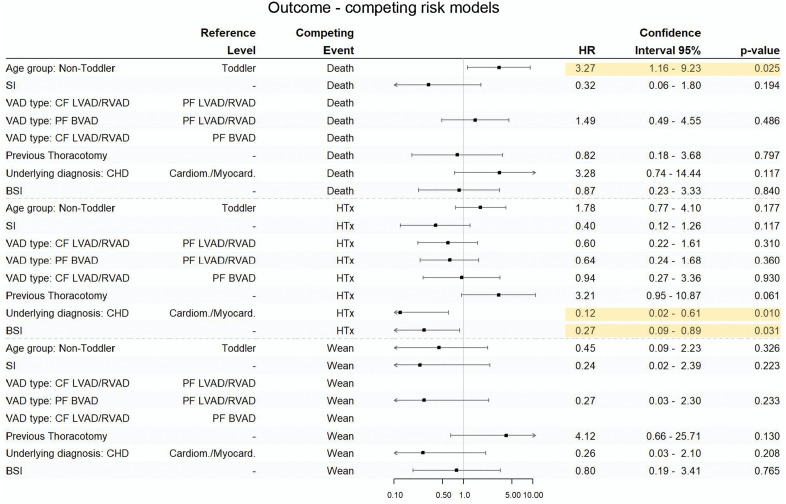
Outcome – competing risk models. Hazard ratios for the competing outcomes death, heart transplantation and weaning from VAD are displayed. SI = superficial percutaneous lead/canula infections; Toddler = < 3 years of age; non-Toddler >3 years of age; Cardiom./Myocard. = Cardiomyopathy/Myocarditis; CHD = Congenital heart defect; CF LVAD = Continuous flow left ventricular assist device; PF BVAD = Pulsatile flow biventricular assist device; PF LVAD/RVAD = Pulsatile flow left/right ventricular assist device; BSI = Bloodstream infection; HTx = Heart transplantation; Wean = Weaning from VAD.

Ten patients (14%) where weaned from VAD, 37 patients (53%) where transplanted, 19 patients (27%) died, and four patients (6%) where still treated on VAD at the end of the study period. Patients older than three years of age had a higher risk for death (HR 3.27; (95% CI 1.16–9.23, p = 0.025)). Patients with CHD as underlying diagnosis (HR 0.12; (95% CI 0.02–0.61, p = 0.010)) and patients with BSI during VAD therapy (HR 0.27; (95% CI 0.09–0.89 p = 0.031)) were less likely transplanted (Fig 5).

Our model reveals no correlation between SI or VAD type and the clinical outcome of pediatric patients treated with a VAD. During variable selection, sex and stroke were dropped from the competing risks model, thus hazard risk was not calculated.

## Discussion

Infectious complications are frequent adverse events in pediatric patients during VAD therapy [28,29]. Among the reported infectious complications SI are frequent and may lead to consecutive complications impacting outcome of VAD therapy in pediatric patients. Therefore, we aimed to evaluate the incidence of SI in pediatric patients on a VAD, to assess the possible risk factors and to determine their impact on overall outcome during VAD-therapy.

In our cohort we detected SI in twelve out of 70 patients with six of them being early and six of them being late infections. This result is in line with previously reported incidence of SI [7,28], although different definitions of local infectious complications make it difficult to compare the studies. Auerbach et al. reported that “External pump component infections” occurred more often late than early [10]. In adults treated with VAD, duration of support is considered a risk factor for infectious complications [30,31]. In line with these results, our observations confirmed the rising risk of SI with long-term VAD support.

Further, we analyzed possible risk factors for SI during VAD therapy including sex, age group, VAD-type, presence of previous thoracotomies, and the underlying diagnosis. Our model did not reveal any of these covariates to be associated with a higher risk of developing a SI, other than being above the age of three at VAD implantation to be associated with an increased risk of SI occurrence and treatment with CF LVAD tends to be protective regarding the occurrence of SI. Since higher mobility is associated with higher infection rates of the cannulas and driveline in adult patients [30,31], we hypothesized that younger children may have higher numbers of SI because they are more mobile and less cooperative. However, in contrast to the aforementioned studies, our model revealed a higher risk for SI in older children compared to younger ones. Interestingly, this was also true regarding the risk for BSI amongs children older than three years of age. According to our model, treatment with a CF LVAD tended to be associated with lower risk of SI and a significant lower risk for BSI. CF LVADs are fully implanted devices with only a single driveline passing the skin, while in PF VAD two (RVAD/LVAD), or four (BVAD) cannulas, exit through the body wall, making them susceptible for SI. Additionally, patients on CF VAD support could be discharged from hospital, protecting them from nosocomial infections. This result is in line with previous findings from the PediMACS registry, that infectious complications were less likely with CF VAD treatment compared to PF VAD treatment [32,33]. Furthermore, according to our model the underlying diagnosis did not influence the risk of SI. In line with this result, Holmann et al. could also not demonstrate a link between underlying diagnosis and infectious complications [34]. From a theoretical point of view, it is reasonable to assume that an SI is a risk factor for systemic infections or BSI. However, SI was not a relevant risk factor for BSI in our model. This may be explained by the fact that wound dressings of cannulas and driveline were done in a standard fashion by trained experts and antimicrobial treatment was guided by institutional infectiology experts (antibiotic stewards). Therefore, potential SI was detected early and, if confirmed, consequently treated helping to prevent subsequent BSI. However, as this is an observational study without a control group for obvious reasons, we cannot provide mechanistic proof for this theory. Nevertheless, we hope to encourage other centers to incorporate specially trained wound-care experts into their teams to improve the management of this highly susceptible population of pediatric VAD patients.

As infections are associated with hypercoagulability [35] we further analyzed if SI led to thromboembolic events in our cohort. Since no patient developed a SI before a stroke, SI could not be included in our CPH model for stroke. This result contrasts with adult patients treated with VAD, in which infectious complications are associated with higher incidence of ischemic strokes [13]. To determine the impact of SI, patient’s age, sex, VAD type, underlying diagnosis, stroke, and the presence of previous thoracotomy on overall outcome (death, heart transplantation, weaning from VAD) a competing risk model was fitted. According to the model, occurrence of SI had no impact on overall outcome. Nevertheless, Auerbach et. al. recently reported that heart transplant patients with prior VAD related infectious were more likely to experience rejection and graft loss [8], highlighting the importance of this topic. In our model, patients older than three years had a higher risk of death during VAD therapy. As noted above, this group was also more more prone to develop a BSI. Given the cohort size and design, the association may reflect chance or residual confounding and merits evaluation in larger, multicenter studies. Further, CHD patients had a HR of 3.3 for mortality relative to patients with cardiomyopathy or myocarditis; however, this result did not reach statistical significance. In line with these results, Peng et. al. also recently reported a higher risk for death when treated with a VAD and CHD as underlying diagnoses [7].

To date, there are only a few studies investigating risk factors for infectious complications in pediatric patients treated on a VAD. Our findings highlight the importance of age-adapted infection prevention strategies in pediatric VAD patients. The trend toward fewer infections with CF devices supports their use when clinically appropriate. Finally, the lack of association between SI and adverse outcomes emphasizes the value of early detection and standardized wound care protocols in preventing complications.

### Limitations

The present study is a single-center retrospective study with a descriptive design and is necessarily modest in size, which may increase the risk of type II errors during hypothesis testing. High variability in the data for some subsets of patients made it difficult to determine whether observed associations of some covariates with outcomes reflected random variation or potentially meaningful patterns. However, pediatric durable VAD implantation is rare worldwide and concentrated in few centers (North America ~130–150 devices/year across ~40 centers [7], Germany: 64 pediatric VAD patients across 8 German centers from 1999–2015 [36]). In comparison, the sample of 70 children presented here represents a larger cohort that may reflect the experience of other specialized centers for the question studied. We mitigated overfitting by restricting adjustment to a small, by medical expertise prespecified set of covariates and emphasize estimation with 95% CIs to communicate uncertainty transparently, though additional confounding caused by other unmeasured factors cannot be excluded. Thus, we refrained from including additional variables in our analyses to reduce the risk of drawing potentially misleading conclusions.

Lastly, the retrospective nature of the study usually introduces inherent limitations, such as reliance on existing clinical routine data, though we were in the fortunate situation of complete data. To improve generalizability, prospective multicenter studies with larger patient cohorts and more detailed stratified microbiological analyses are warranted.

## Conclusion

Superficial percutaneous lead/canula infections occurred frequently during VAD therapy in children, without leading to higher rates of bloodstream infections or mortality. Being supported on CF LVAD seem to be associated with a reduced risk of superficial percutaneous lead/canula infections and VAD implantation above the age of three years seem to be associated with an increased risk of SI and BSI, while underlying diagnosis and previous thoracotomy were not associated with an elevated risk. Further studies are warranted to determine the impact of infectious complications on overall clinical outcome and to optimize surveillance and clinical management of pediatric patients treated on VAD.

## References

[1] Adachi I, Khan MS, Guzmán-Pruneda FA, Fraser CD 3rd, Mery CM, Denfield SW, et al. Evolution and impact of ventricular assist device program on children awaiting heart transplantation. Ann Thorac Surg. 2015;99(2):635–40. doi: 10.1016/j.athoracsur.2014.10.010 25530089

[2] Fraser CD Jr, Jaquiss RDB, Rosenthal DN, Humpl T, Canter CE, Blackstone EH, et al. Prospective trial of a pediatric ventricular assist device. N Engl J Med. 2012;367(6):532–41. doi: 10.1056/NEJMoa1014164 22873533

[3] Rossano JW, Dipchand AI, Edwards LB, Goldfarb S, Kucheryavaya AY, Levvey Rn BJ, et al. The registry of the International society for heart and lung transplantation: nineteenth pediatric heart transplantation report-2016; focus theme: primary diagnostic indications for transplant. J Heart Lung Transplant. 2016;35(10):1185–95. doi: 10.1016/j.healun.2016.08.018 27772670

[4] Hetzer R, Stiller B. Technology insight: Use of ventricular assist devices in children. Nat Clin Pract Cardiovasc Med. 2006;3(7):377–86. doi: 10.1038/ncpcardio0575 16810173

[5] Aaronson KD, Slaughter MS, Miller LW, McGee EC, Cotts WG, Acker MA, et al. Use of an intrapericardial, continuous-flow, centrifugal pump in patients awaiting heart transplantation. Circulation. 2012;125(25):3191–200. doi: 10.1161/CIRCULATIONAHA.111.058412 22619284

[6] Rohde S, van Puyvelde J, Veen KM, Schweiger M, Biermann D, Amodeo A, et al. The European registry for patients with mechanical circulatory support (EUROMACS): fourth paediatric EUROMACS (Paedi-EUROMACS) report. Eur J Cardiothorac Surg. 2024;66(2):ezae276. doi: 10.1093/ejcts/ezae276 39029919

[7] Peng DM, Davies RR, Simpson KE, Shugh SB, Morales DLS, Jacobs JP, et al. Seventh annual society of thoracic surgeons pedimacs report. Ann Thorac Surg. 2024;117(4):690–703. doi: 10.1016/j.athoracsur.2023.11.035 38123046

[8] Auerbach SR, Cantor RS, Bradford TT, Bock MJ, Skipper ER, Koehl DA, et al. The effect of infectious complications during ventricular assist device use on outcomes of pediatric heart transplantation. ASAIO J. 2022;68(2):287–96. doi: 10.1097/MAT.0000000000001442 34264872

[9] Goldstein DJ, Naftel D, Holman W, Bellumkonda L, Pamboukian SV, Pagani FD, et al. Continuous-flow devices and percutaneous site infections: clinical outcomes. J Heart Lung Transplant. 2012;31(11):1151–7. doi: 10.1016/j.healun.2012.05.004 22766022

[10] Auerbach SR, Richmond ME, Schumacher KR, Lopez-Colon D, Mitchell MB, Turrentine MW, et al. Infectious complications of ventricular assist device use in children in the United States: data from the pediatric interagency registry for mechanical circulatory support (Pedimacs). J Heart Lung Transplant. 2018;37(1):46–53. doi: 10.1016/j.healun.2017.09.013 29107545 PMC5849428

[11] Gordon RJ, Quagliarello B, Lowy FD. Ventricular assist device-related infections. Lancet Infect Dis. 2006;6(7):426–37. doi: 10.1016/S1473-3099(06)70522-9 16790383

[12] Nienaber J, Wilhelm MP, Sohail MR. Current concepts in the diagnosis and management of left ventricular assist device infections. Expert Rev Anti Infect Ther. 2013;11(2):201–10. doi: 10.1586/eri.12.163 23409825

[13] Frontera JA, Starling R, Cho S-M, Nowacki AS, Uchino K, Hussain MS, et al. Risk factors, mortality, and timing of ischemic and hemorrhagic stroke with left ventricular assist devices. J Heart Lung Transplant. 2017;36(6):673–83. doi: 10.1016/j.healun.2016.12.010 28110971

[14] Jordan LC, Ichord RN, Reinhartz O, Humpl T, Pruthi S, Tjossem C, et al. Neurological complications and outcomes in the Berlin Heart EXCOR® pediatric investigational device exemption trial. J Am Heart Assoc. 2015;4(1):e001429. doi: 10.1161/JAHA.114.001429 25613996 PMC4330068

[15] Miera O, Schmitt KRL, Delmo-Walter E, Ovroutski S, Hetzer R, Berger F. Pump size of Berlin Heart EXCOR pediatric device influences clinical outcome in children. J Heart Lung Transplant. 2014;33(8):816–21. doi: 10.1016/j.healun.2014.03.007 24836553

[16] Kormos RL, Antonides CFJ, Goldstein DJ, Cowger JA, Starling RC, Kirklin JK, et al. Updated definitions of adverse events for trials and registries of mechanical circulatory support: A consensus statement of the mechanical circulatory support academic research consortium. J Heart Lung Transplant. 2020;39(8):735–50. doi: 10.1016/j.healun.2020.03.010 32386998

[17] Andersen PK, Gill RD. Cox’s regression model for counting processes: a large sample study. Ann Statist. 1982;10(4). doi: 10.1214/aos/1176345976

[18] Akaike H. Information Theory and an extension of the maximum likelihood principle. In: Springer Series in Statistics. Springer New York; 1998. 199–213. doi: 10.1007/978-1-4612-1694-0_15

[19] Burnham KP, Anderson DR. Multimodel inference. Sociol Methods Res. 2004;33(2):261–304. doi: 10.1177/0049124104268644

[20] Gordon M, Lumley T. Forestplot: Advanced forest plot using “grid” graphics. 2023. https://CRAN.R-project.org/package=forestplot

[21] Kassambara A, Kosinski M, Biecek P. Survminer: drawing survival curves using “ggplot2.” 2021. https://CRAN.R-project.org/package=survminer

[22] Lüdecke D. sjPlot: Data visualization for statistics in social science. https://CRAN.R-project.org/package=sjPlot

[23] Stevenson M, Sergeant E. epiR: tools for the analysis of epidemiological data. 2024.

[24] Therneau TM. A package for survival analysis in R. 2024.

[25] Wickham H, Averick M, Bryan J, Chang W, McGowan L, François R, et al. Welcome to the Tidyverse. JOSS. 2019;4(43):1686. doi: 10.21105/joss.01686

[26] Wreede LC de, Fiocco M, Putter H. mstate: AnRPackage for the analysis of competing risks and multi-state models. J Stat Soft. 2011;38(7). doi: 10.18637/jss.v038.i07

[27] Yoshida K, Bartel A. tableone: Create “Table 1” to describe baseline characteristics with or without propensity score weights. 2022. https://CRAN.R-project.org/package=tableone

[28] Rosenthal DN, Almond CS, Jaquiss RD, Peyton CE, Auerbach SR, Morales DR, et al. Adverse events in children implanted with ventricular assist devices in the United States: data from the pediatric interagency registry for mechanical circulatory support (PediMACS). J Heart Lung Transp. 2016;35: 569–77. doi: 10.1016/j.healun.2016.03.005 PMC5113942 27197775

[29] Auerbach SR, Richmond ME, Schumacher KR, Lopez-Colon D, Mitchell MB, Turrentine MW, et al. Infectious complications of ventricular assist device use in children in the United States: data from the pediatric interagency registry for mechanical circulatory support (Pedimacs). J Heart Lung Transplant. 2018;37(1):46–53. doi: 10.1016/j.healun.2017.09.013 29107545 PMC5849428

[30] Sharma V, Deo SV, Stulak JM, Durham LA 3rd, Daly RC, Park SJ, et al. Driveline infections in left ventricular assist devices: implications for destination therapy. Ann Thorac Surg. 2012;94(5):1381–6. doi: 10.1016/j.athoracsur.2012.05.074 22818961

[31] Zierer A, Melby SJ, Voeller RK, Guthrie TJ, Ewald GA, Shelton K, et al. Late-onset driveline infections: the Achilles’ heel of prolonged left ventricular assist device support. Ann Thorac Surg. 2007;84(2):515–20. doi: 10.1016/j.athoracsur.2007.03.085 17643627

[32] Blume ED, VanderPluym C, Lorts A, Baldwin JT, Rossano JW, Morales DLS, et al. Second annual pediatric interagency registry for mechanical circulatory support (Pedimacs) report: pre-implant characteristics and outcomes. J Heart Lung Transplant. 2018;37(1):38–45. doi: 10.1016/j.healun.2017.06.017 28965736

[33] Rosenthal DN, Almond CS, Jaquiss RD, Peyton CE, Auerbach SR, Morales DR, et al. Adverse events in children implanted with ventricular assist devices in the United States: data from the pediatric interagency registry for mechanical circulatory support (PediMACS). J Heart Lung Transplant. 2016;35: 569–77. doi: 10.1016/j.healun.2016.03.005 27197775 PMC5113942

[34] Holman WL, Kirklin JK, Naftel DC, Kormos RL, Desvign-Nickens P, Camacho MT, et al. Infection after implantation of pulsatile mechanical circulatory support devices. J Thorac Cardiovasc Surg. 2010;139(6):1632-1636.e2. doi: 10.1016/j.jtcvs.2010.01.014 20363482

[35] Byrnes JW, Bhutta AT, Rettiganti MR, Gomez A, Garcia X, Dyamenahalli U, et al. Steroid therapy attenuates acute phase reactant response among children on ventricular assist device support. Ann Thorac Surg. 2015;99(4):1392–8. doi: 10.1016/j.athoracsur.2014.11.046 25669667

[36] Lammers AE, Sprenger KS, Diller G-P, Miera O, Lebherz C, Helm PC, et al. Ventricular assist devices in paediatric cardiomyopathy and congenital heart disease: an analysis of the German National Register for Congenital Heart Defects. Int J Cardiol. 2021;343:37–44. doi: 10.1016/j.ijcard.2021.08.047 34487787

